# Using a Novel Pulsed Field Ablation Technique to Identify the Critical Isthmus Within a Tachycardia Circuit

**DOI:** 10.19102/icrm.2025.16023

**Published:** 2025-02-15

**Authors:** James Mannion, Faizan Rathore, Jakub David, Jonathan Lyne

**Affiliations:** 1 Electrophysiology Department, Beacon Hospital, Sandyford, Dublin, Ireland; 2 Cardiology Department, Midlands Regional Hospital Mullingar, Mullingar, Westmeath, Ireland; 3 Medtronic Ireland, Dublin, Ireland; 4 Technical University of Liberec, Liberec, Czech Republic; 5 School of Medicine, University College Dublin, Belfield, Dublin, Ireland

**Keywords:** Atrial tachycardia, catheter ablation, novel mapping technique, pulsed field ablation, reversible electroporation

## Abstract

Pulsed field ablation uses irreversible electroporation to interrupt cellular membranes and induce myocyte apoptosis. Reversible electroporation has been used in other specialties, but its utility in cardiac ablation is unknown. Here, a 69-year-old woman undergoing repeat ablation for atypical atrial flutter presented with extensive scar after cardiac surgery (including MAZE) and previous ablation, leading to a macro re-entry circuit. To minimize superfluous lesions and further arrhythmia, we used a single pulse confirming the isthmus location, with cessation of the arrhythmia. As a conclusion, reversible electroporation may be used to test areas of interest prior to irreversible lesion creation.

## Introduction

Pulsed field ablation (PFA) is an emerging non-thermal ablation technology that uses electroporation to interrupt cellular membranes and induce apoptosis.^1^ PFA has been demonstrated to be non-inferior to conventional thermal energy in procedural efficacy and freedom from atrial fibrillation, suggesting it achieves lesion durability comparable to conventional thermal energy.^2^

At a cellular level, PFA is potentially more cardioselective than conventional thermal energy, demonstrated by the absence of esophageal lesions on necropsy in porcine models. This was in comparison with radiofrequency, where identifiable esophageal lesions were observed post-ablation.^3^

Current evidence suggests that PFA lesions are typically durable, with persistence of pulmonary vein isolation (PVI) and no change in low-voltage areas when sequential voltage maps were analyzed in 20 patients.^4^ However, the concept of “reversible” PFA (PF-rev) lesions has been demonstrated in a recent study when abbreviated PFA (delivery of a single pulse of PF energy, compared to therapeutic PULSE3™ [Medtronic, Minneapolis, MN, USA] delivery that consists of 1500 pulses) was applied to atrial tissue.^5^ Application of PFA in this model demonstrated measurable conduction prolongation (P–R prolongation and transient atrioventricular [AV] block) and, upon subsequent histological examination of the myocardial tissue, either trace or no fibrosis was present.^4^ Hence, this may prove to be of utility in identifying targets of ablation prior to modifying the arrhythmia substrate.

In this case, we demonstrate the novel use of low-intensity PFA application to identify a critical isthmus by delaying and eventually terminating the arrhythmia and restoring sinus rhythm, prior to undertaking irreversible electroporation in a patient undergoing mapping and ablation for atypical atrial flutter.

## Case presentation

A 69-year-old woman initially presented with worsening dyspnea and palpitations, with a background history of PVI, mitral valve repair, and MAZE procedure. Postoperatively, she suffered from recurrent tachycardia episodes despite medical therapy and cardioversion, so she proceeded to have an electrophysiological (EP) study.

The study at the time identified the arrhythmia as left-sided roof-dependent atrial flutter. Ablation across the roof terminated the tachycardia back to sinus rhythm. PVI was performed at the same time on the left-sided veins, which were found to have reconnected.

On this occasion, she presented with recurrent palpitations, and an electrocardiogram (ECG) confirmed the recurrence of atrial arrhythmia. We proceeded to complete another EP study.

Eccentric, left-sided activation was noted during the second EP study, and access to the left atrium was obtained transeptally under transesophageal echo guidance. Activation and voltage mapping were undertaken using a high-density mapping and ablation lattice tip catheter (Sphere-9 catheter, Affera™ Mapping and Ablation System; Medtronic).

**Figure 1:**
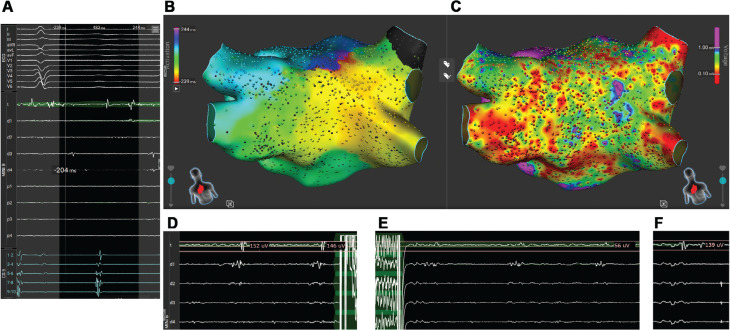
**A:** Intracardiac electrograms at the channel. **B:** Local activation time map in atrial tachycardia. **C:** Bipolar voltage map in atrial tachycardia. **D:** Intracardiac electrogram amplitude before short pulse delivery (during atrial tachycardia). **E:** Intracardiac electrogram showing amplitude after short pulse delivery (during atrial tachycardia). **F:** Intracardiac electrogram showing amplitude after 35 min (during proximal coronary sinus pacing).

A recurrent left-sided roof-dependent flutter was observed, with fractionated electrograms along the atrial roof. To identify the ablation target, very-short-duration PFA consisting of a single pulse was conducted **(Figure 1)**.

**Figure 2:**
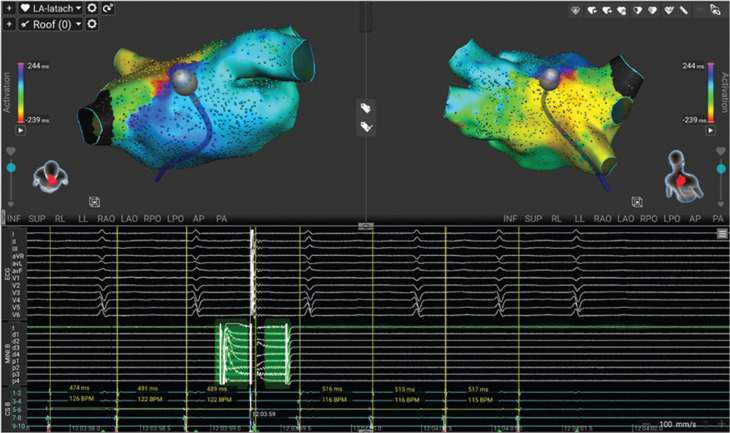
A single application of pulsed field energy to confirm the area of interest, with evident cessation of the re-entry tachycardia. We found a potential reduction in local voltage with prolongation in cycle length followed by restoration of sinus rhythm.

Following the application, tachycardia cycle length prolonged and terminated with restoration of sinus rhythm **(Figure 2)**, confirming the critical isthmus using the PF-rev technique. The location was remapped with an interval of 10 min and demonstrated minimal change in endocardial voltages. A durable lesion set was then created using standard PFA application with an endpoint of a successful line of block on the roof, and attempts to reinduce flutter at the end of the case were unsuccessful.

## Discussion

The pattern of myocardial recovery following PF-rev application has been described in human hearts,^3,5,6^ but its utility in identifying critical isthmus has been demonstrated in only two male patients.^3^ Irreversible electroporation is used in standard PFA ablation to create robust lesions via disruption of the cellular membrane and induce apoptosis.^6^ These are usually deployed in a sequence of several pulses. Chaigne et al., however, observed that single electrical pulses transiently influence calcium homeostasis and allow for subsequent recovery of cardiac myocytes.^6^ This observation has been reported in previous defibrillation trials and is termed myocardial “stunning.”^7,8^ In addition to cardiac trials, PF-rev itself has been used in other medical specialities.^9,10^

Interest in using PF-rev in cardiac ablation is expanding exponentially. In rodent hearts, single pulses of PF-rev resulted in rapid alterations of intracellular calcium, proportional to the voltage delivered. Spontaneous contractions of sarcomeres were observed after delivering non-lethal doses of pulsed energy, which progressively reduced over 20 min.^6^

Koruth et al. have demonstrated the use of PF-rev in human atrial myocardium, with a clear reduction (up to 69.9%) in local voltage of bipolar voltage maps immediately after PF-rev with marked recovery post-application. Measured voltages returned to 84% of baseline by 3 min.^5^ Other transient observations included P–R prolongation and AV block if energy was delivered in proximity to the AV node. Hence, this technique has the potential to become a novel tool to identify ablation targets prior to undertaking irreversible electroporation and creation of further scar, especially in a case like ours where multiple prior interventions resulted in extensive arrhythmogenic substrate.

The complexity of cardiac ablation procedures continues to evolve with a higher volume of patients needing repeated procedures, an increasing number of patients with congenital heart disease surviving into adulthood, and reductions in mortality post-myocardial infarction or cardiac surgery.^11^ The arrhythmogenic substrate in these populations adds a dimension of complexity far beyond the classic anatomical ablation model. Mapping and identifying critical isthmuses are integral to treating these patients with complex arrhythmias. The ability to test a location with a short-duration PF-rev without committing to a transmural lesion and adding to the total arrhythmogenic scar burden^12^ could be an invaluable tool in the arsenal of electrophysiologists. The technique currently requires the delivery of focal or large focal ablation, which may not be applicable to other PFA platforms.
